# Retrospective evaluation of echocardiographic variables for prediction of heart failure hospitalization in heart failure with preserved versus reduced ejection fraction: A single center experience

**DOI:** 10.1371/journal.pone.0244379

**Published:** 2020-12-22

**Authors:** Michael M. Hammond, Changyu Shen, Stephanie Li, Dhruv S. Kazi, Marwa A. Sabe, A. Reshad Garan, Lawrence J. Markson, Warren J. Manning, Allan L. Klein, Sherif F. Nagueh, Jordan B. Strom

**Affiliations:** 1 Department of Biostatistics, Boston University School of Public Health, Boston, MA, United States of America; 2 Division of Cardiovascular Medicine, Richard A. and Susan F. Smith Center for Outcomes Research in Cardiology, Beth Israel Deaconess Medical Center, Boston, MA, United States of America; 3 Division of Cardiovascular Medicine, Department of Medicine, Beth Israel Deaconess Medical Center, Boston, MA, United States of America; 4 Harvard Medical School, Boston, MA, United States of America; 5 Center for Healthcare Delivery Science, Beth Israel Deaconess Medical Center, Boston, MA, United States of America; 6 Information Systems, Beth Israel Deaconess Medical Center, Boston, MA, United States of America; 7 Department of Radiology, Beth Israel Deaconess Medical Center, Boston, MA, United States of America; 8 The Robert and Suzanne Tomsich Department of Cardiovascular Medicine, Cleveland Clinic, Cleveland Clinic Lerner College of Medicine, Cleveland, OH, United States of America; 9 Department of Cardiology, Houston Methodist Hospital, Weill Cornell Medical College, Houston, TX, United States of America; Scuola Superiore Sant'Anna, ITALY

## Abstract

**Background:**

Limited data exist on the differential ability of variables on transthoracic echocardiogram (TTE) to predict heart failure (HF) readmission across the spectrum of left ventricular (LV) systolic function.

**Methods:**

We linked 15 years of TTE report data (1/6/2003-5/3/2018) at Beth Israel Deaconess Medical Center to complete Medicare claims. In those with recent HF, we evaluated the relationship between variables on baseline TTE and HF readmission, stratified by LVEF.

**Results:**

After excluding TTEs with uninterpretable diastology, 5,900 individuals (mean age: 76.9 years; 49.1% female) were included, of which 2545 individuals (41.6%) were admitted for HF. Diastolic variables augmented prediction compared to demographics, comorbidities, and echocardiographic structural variables (p < 0.001), though discrimination was modest (c-statistic = 0.63). LV dimensions and eccentric hypertrophy predicted HF in HF with reduced (HFrEF) but not preserved (HFpEF) systolic function, whereas LV wall thickness, NT-proBNP, pulmonary vein D- and Ar-wave velocities, and atrial dimensions predicted HF in HFpEF but not HFrEF (all interaction p < 0.10). Prediction of HF readmission was not different in HFpEF and HFrEF (p = 0.93).

**Conclusions:**

In this single-center echocardiographic study linked to Medicare claims, left ventricular dimensions and eccentric hypertrophy predicted HF readmission in HFrEF but not HFpEF and left ventricular wall thickness predicted HF readmission in HFpEF but not HFrEF. Regardless of LVEF, diastolic variables augmented prediction of HF readmission compared to echocardiographic structural variables, demographics, and comorbidities alone. The additional role of medication adherence, readmission history, and functional status in differential prediction of HF readmission by LVEF category should be considered for future study.

## Introduction

Heart failure (HF) is a significant public health problem accounting for nearly 1 million hospitalizations annually in developed countries with estimates expected to increase by >8 million people in the US by 2030, accounting for nearly $70 billion in costs [1–6]. Despite the frequency of HF hospitalization, prediction of individuals at high risk for future rehospitalization remains difficult using traditional approaches [7]. While many echocardiographic variables such as left ventricular (LV) wall thickness, mass, and chamber sizes, have been associated with an adverse prognosis in individuals with prior heart failure hospitalization [8], only a few studies have evaluated the ability of these variables to predict HF readmission in a mixed systolic function cohort [9–23]. Thus, whether there is a significant interaction between LV systolic function category and the relative importance of individual echocardiographic variables in driving HF readmission risk remains unclear. Furthermore, whether competing risk of mortality may impact the predictive ability of HF readmission models differentially across LV functional categories remains uncertain.

As such, we conducted a retrospective cohort study using a large database of structured echocardiographic report data from transthoracic echocardiograms (TTEs) performed over 18 years at Beth Israel Deaconess Medical Center linked to complete Medicare inpatient claims to evaluate if 1) individual variables and diastolic functional variables as a whole differ in their ability to predict HF readmission in individuals with HF with reduced (HFrEF) vs. preserved (HFpEF) and 2) whether the competing risk of mortality impacts predictive accuracy differently in HFrEF vs. HFpEF. We hypothesized that individual variables would differ in their ability to predict HF readmissions in HFpEF and HFrEF.

## Materials and methods

### Study population

Structured echocardiographic report data from 167,368 TTEs on 75,681 individuals ≥65 years old, 1/1/2000-9/20/2018, at Beth Israel Deaconess Medical Center (BIDMC) were directly linked to 100% Medicare inpatient discharge claims, 2003–2017, in the Medicare Provider Analysis and Review dataset. The Medicare Provider Analysis and Review dataset represents a complete sample of Part A hospitalization discharge claims on Medicare Fee-for-service beneficiaries and has been used extensively to study health outcomes [24–27]. Only the first sequential TTE was considered for the current analysis.

In order to identify a cohort of patients with recent HF hospitalization, the sample was restricted to individuals with at least one claim for HF hospitalization whose discharge date was within 1-year prior to the index TTE. HF hospitalization was defined as having International Classification of Diseases, 9^th^ Revision, Clinical Modification (ICD-9-CM) codes 428.X (prior to October 1, 2015) or International Classification of Diseases, 10^th^ Revision, Clinical Modification (ICD-10-CM) codes I50.X (after October 1, 2015), in either the first or second position. We excluded echocardiographic studies that included a mitral prosthesis, annuloplasty ring, MitraClip, moderate or greater mitral annular calcification, evidence of endocarditis, pacing or conduction abnormality, pericardial constriction, greater than moderate mitral regurgitation, mild or greater mitral stenosis, or greater than moderate aortic regurgitation [20]. Studies using the linked ENCOR-Medicare dataset have been approved by the Institutional Review Board at Beth Israel Deaconess Medical Center with a waiver of informed consent. Data access is restricted by according to current Medicare data use agreements.

### Covariates

The predictor variables included demographic, physiologic, echocardiographic structural variables and diastolic functional variables and clinical comorbidities. Demographic and physiologic variables included age, sex, blood pressure, heart rate, height, weight, body mass index, and inpatient/outpatient status. Echocardiographic structural and functional variables were LV ejection fraction (LVEF), LV systolic and diastolic linear dimensions, LV septal and posterior wall thickness, relative wall thickness, left ventricular mass, right atrial superoinferior linear dimension, right ventricular basal diastolic dimension, peak transvalvular aortic velocity, and severity of aortic, mitral, and tricuspid regurgitation. Left ventricular ejection fraction was obtained via the Simpson’s biplane method of disks or three-dimensional volumetric methods when feasible.

Echocardiographic diastolic variables included mitral valve peak E-wave velocity, mitral valve peak A-wave velocity, tissue Doppler lateral and septal e’ velocities, pulmonary vein S, D, and Ar velocities, and peak tricuspid regurgitant pressure gradient by the modified Bernoulli equation. Additionally, the E/A, S/D, and E/e’ ratios were determined based on taking the ratio of the individual components. Left ventricular hypertrophy was defined was defined as a left ventricular mass index > 95 g/m^2^ for females and > 115 g/m^2^ for males [20]. Concentric remodeling was defined as a relative wall thickness >0.42 and eccentric remodeling as a relative wall thickness ≤ 0.42 [20]. All these variables were obtained during the index TTE. Diastolic grade was inferred retrospectively using the convention established in the American Society of Echocardiography 2016 guidelines [20]. Specifically, four parameters (septal e’ < 7 cm/s or lateral e’ < 10 cm/s, average E/e’ > 14, peak tricuspid regurgitant velocity> 2.8 m/s, and left atrial volume index > 34 mL/m^2^) were assessed in those with LVEF ≥ 50% [20]. If <50% were positive, the TTE was determined to have normal diastolic function. If > 50% were positive, the TTE was determined to have diastolic dysfunction and if 50% were positive, the TTE was determined to have indeterminate diastolic function [20]. Amongst those with an LVEF < 50% or diastolic dysfunction by the prior schema, three parameters were evaluated to assess LV filling patterns: average E/e’ > 14, peak tricuspid regurgitant velocity > 2.8 m/s, and left atrial volume index > 34 mL/m^2^ [20]. If the E/A ratio was ≥2.0, the designation of Grade III diastolic dysfunction was applied. If E/A was ≤ 0.8 and E-wave velocity ≤ 50 cm/s or if ≥ 2/3 of parameters were negative, the designation of Grade I diastolic dysfunction was applied. If ≥ 2/3 of the parameters were positive, the designation of Grade II diastolic dysfunction was applied. Otherwise, the diastolic function was considered to be indeterminate. Grade II and III diastolic dysfunction were combined in analyses.

Clinical comorbidities were determined based on the Elixhauser Comorbidity Index using hospital discharge claims within the year prior to the index echocardiogram to define comorbidity variables. The Elixhauser Comorbidity Index is used to define 29 comorbidity variables as well as a composite comorbidity score based on the count of these comorbidity variables for a given individual, and has been used extensively previously in the analysis of administrative data to control for confounding by clinical variables [28]. Additionally, where performed, the most recent N-terminal pro-brain natriuretic peptide (NT-proBNP) value prior to the index TTE was extracted from BIDMC clinical databases.

### Outcomes

The primary outcome was any HF readmission whose admission date was within one year after the date of the index TTE. HF readmission was defined as any ICD-9-CM 428.X code (prior to October, 1, 2015) or ICD-10-CM I50.X code (after October 1, 2015) in the first or second position. For individuals with multiple HF readmissions meeting this definition, only the first was considered. If individuals were admitted for HF at the time of their index TTE, only subsequent hospitalizations (i.e. occurring at least 1 day after the date of discharge for the hospitalization in which the TTE was performed) were considered.

### Statistical analysis

Analyses were stratified by presence (HFpEF; LVEF ≥50%) or absence (HFrEF; LVEF < 50%) of preserved left ventricular systolic function. We summarized the characteristics of individuals by presence or absence of hospitalization. Baseline clinical and echocardiographic characteristics of included individuals are reported as means and standard deviations (SDs), medians and interquartile ranges (IQRs), or frequencies and percentages and compared between those with and without HF hospitalizations within a year of the index TTE using t-tests or Wilcoxon rank sum tests for continuous variables and Fisher’s exact or Chi-Squared tests for categorical variables.

Due to different measurement units and variability, all continuous predictors were standardized to enable comparison. Logistic regression models were used to determine the univariate odds ratios for HF hospitalization at 1-year for a 1-standard deviation increase in continuous predictors or a 1-unit increase in categorical predictors, stratified by presence or absence of a preserved LVEF. Multiplicative interaction was evaluated between each baseline variable and risk of HF readmission within 1-year by presence or absence of preserved LVEF using a p-value threshold < 0.10 to define significance.

Subsequently, to evaluate if echocardiographic variables augment prediction of HF hospitalization, the study population was randomly split into a 50% derivation and validation sample. The c-statistics for both derivation and validation samples were determined using nested multivariable logistic regression models containing all demographic and physiologic variables (Model 1), demographic and physiologic variables plus comorbidities (Model 2), demographic, physiologic variables, comorbidities, and echocardiographic structural variables (Model 3), and demographic, physiologic variables, comorbidities, echocardiographic structural variables, and diastolic functional variables (Model 4). The adjusted odds ratios, 95% confidence intervals, and p-values for significant variables in Model 4 are reported. Receiver operator characteristic (ROC) curves were created for each model and the areas under the curve (AUCs) compared using the DeLong test [29].

Subsequently, the model comparison was repeated, stratified by presence or absence of a preserved LVEF at baseline. To assess if the fully augmented model (i.e. Model 4) discriminated HF readmission better in the HFrEF vs. HFpEF subgroup, a two sample t-test was used to compare the optimism-adjusted AUCs.

In order to account for the competing risk of death, sensitivity analyses were performed using a composite outcome of death or HF readmission within 1-year from the index TTE. Statistical analyses were performed using SAS v9.4 or JMP Pro v 13.0 (SAS Institute, Cary, NC) using a two-tailed p < 0.05.

## Results

**Fig 1:**
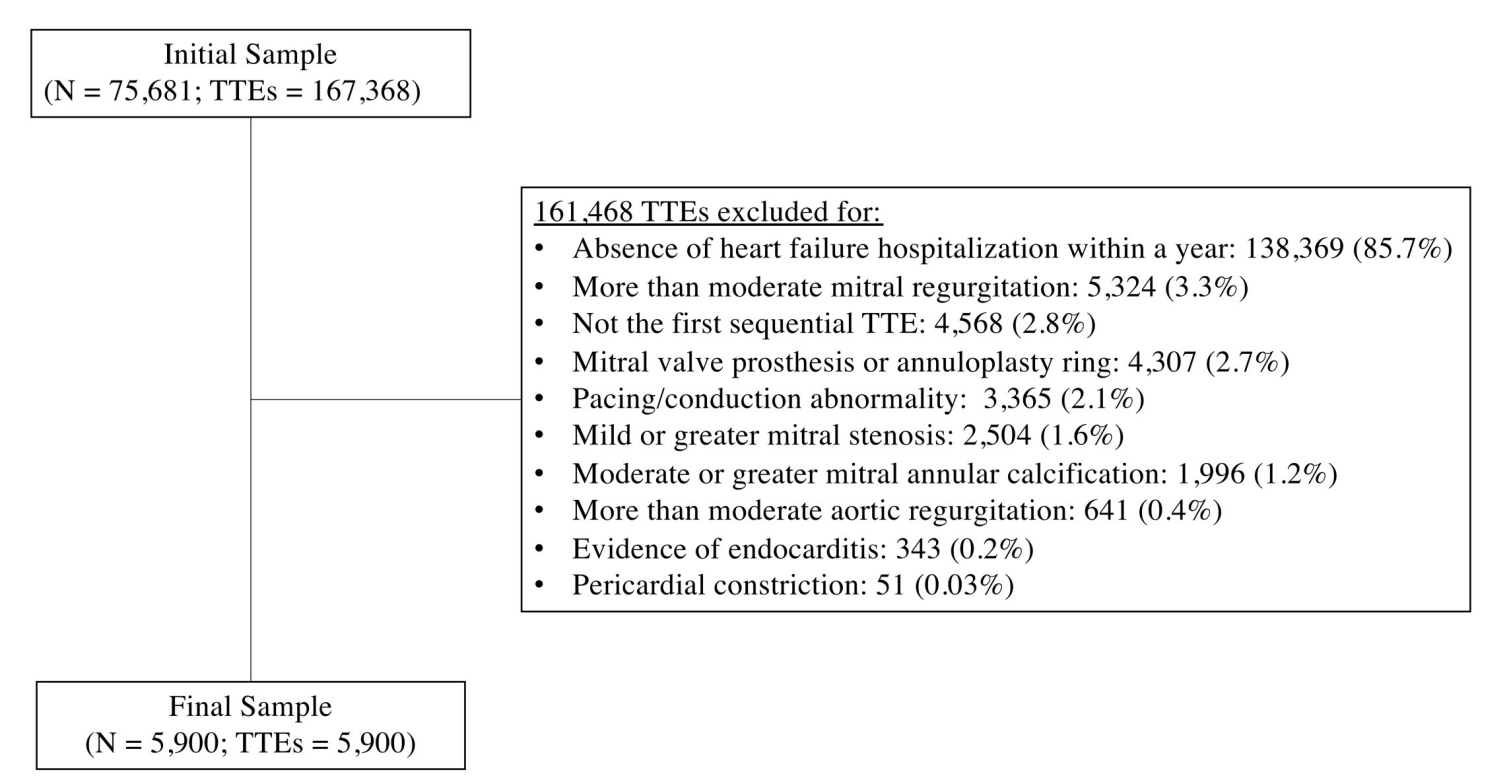
Flow diagram of patients included in study. TTEs = transthoracic echocardiograms.

Of 167,368 TTEs on 75,681 individuals initially considered for inclusion, 5,900 TTEs on 5,900 individuals were included after exclusions (**Fig 1**). The overall cohort had a mean age of 76.9 ± 10.9 years, mean LVEF of 52.1% ± 20.3%, and 49.1% were female.

Of those included, 2438 (41.3%) had an LVEF < 50% and 3462 (58.9%) had an LVEF ≥ 50% (**Table 1**). Individuals with HFrEF were less frequently female, had higher NT-proBNP measurements, larger LV dimensions, greater overall LV hypertrophy and particularly eccentric hypertrophy, more severe mitral and tricuspid regurgitation, and fewer overall comorbidities (all p < 0.05).

**Table 1:** Baseline characteristics of included individuals by left ventricular function category^a^.

Variable	N obs	Heart Failure with Reduced Ejection Fraction (N = 2438)	Heart Failure with Preserved Ejection Fraction (N = 3462)	p-value
**Demographics and Physiologic Variables**				
Age (years)	5900	76.3 ± 10.5	77.4 ± 11.2	< 0.001
Female–no. (%)	5900	871 (35.7)	2023 (58.4)	< 0.001
Systolic BP (mmHg)	5763	123 ± 41	129 ± 27	< 0.001
Diastolic BP (mmHg)	5751	68 ± 34	68 ± 39	0.93
Heart rate (bpm)	5447	77 ± 17	75 ± 21	< 0.001
Height (cm)	5657	169.3 ± 11.4	165.2 ± 11.2	< 0.001
Weight (kg)	5731	79.6 ± 21.2	82.0 ± 25.3	< 0.001
Body Mass Index–(kg/m^2^)	5628	28.1 ± 15.5	29.9 ± 8.5	< 0.001
Inpatient at the time of echocardiogram–no. (%)	5900	1904 (78.2)	2625 (75.8)	0.04
NT-proBNP–median (IQR)	3142	6410 (2605–14935)	2487 (1034–7336)	< 0.001
**Echocardiographic Diastolic Function Variables**				
Mitral valve peak E-wave velocity (m/s)	5102	0.9 ± 0.3	1.0 ± 0.4	< 0.001
Mitral valve peak A wave velocity (m/s)	3800	0.8 ± 0.4	0.9 ± 0.3	< 0.001
Mitral valve E/A ratio	3795	1.4 ± 1.0	1.3 ± 0.8	< 0.001
Mitral valve deceleration time (ms)	4866	194 ± 69	225 ± 75	< 0.001
Lateral Mitral e’ Velocity (cm/s)	2969	7.7 ± 12.2	9.3 ± 21.8	0.009
Septal Mitral e’ Velocity (cm/s)	2931	5.5 ± 6.1	6.8 ± 12.2	< 0.001
Pulmonary vein S wave velocity (m/s)	1418	0.5 ± 0.2	0.6 ± 0.5	< 0.001
Pulmonary vein D wave velocity (m/s)	1298	0.5 ± 0.5	0.5 ± 0.3	0.75
Pulmonary vein S/D ratio	1292	1.1 ± 0.6	1.3 ± 1.1	0.005
Pulmonary vein Ar wave velocity (m/s)	764	0.3 ± 0.1	0.3 ± 0.3	0.003
E/e’ ratio	2855	15.8 ± 7.1	14.1 ± 6.2	< 0.001
Left atrial size (cm)				
Superoinferior	5042	5.9 ± 0.9	5.8 ± 0.9	< 0.001
Anteroposterior	5248	4.5 ± 0.8	4.4 ± 0.8	< 0.001
Left atrial volume index (cm^3^/m^2^)	551	39.4 ± 11.9	38.1 ± 11.0	0.19
Peak tricuspid regurgitant gradient (mmHg)	4509	36 ± 12	37 ± 14	0.02
Presence of Diastolic Dysfunction–no. (%)	5898	N/A		N/A
Diastolic dysfunction			439 (12.7)	
Indeterminate			586 (16.9)	
Normal			2437 (70.4)	
Diastolic Grade–no. (%)	5898			0.03
Indeterminate		129 (5.3)	94 (2.7)	
Normal/Grade I		2061 (84.5)	3065 (88.5)	
Grade II		153 (6.3)	218 (6.3)	
Grade III		93 (3.8)	85 (2.5)	
**Echocardiographic Structural Variables**				
Left ventricular ejection fraction (%)	5900	31.8 ± 10.2	66.5 ± 11.4	< 0.001
Left ventricular systolic dimension (cm)	3039	4.4 ± 1.0	2.8 ± 0.6	< 0.001
Left ventricular diastolic dimension (cm)	5238	5.3 ± 0.9	4.4 ± 0.7	< 0.001
Left ventricular septal wall thickness (cm)	5193	1.1 ± 0.2	1.2 ± 0.2	< 0.001
Left ventricular posterior wall thickness (cm)	5174	1.1 ± 0.2	1.2 ± 0.2	< 0.001
Relative wall thickness	5132	0.43 ± 0.13	0.55 ± 0.16	< 0.001
Left ventricular mass (g)	5131	244.6 ± 78.9	201.6 ± 25.5	< 0.001
Left ventricular mass index (g/m^2^)	4965	128.3 ± 40.0	106.0 ± 32.5	< 0.001
Left ventricular hypertrophy–no. (%)	5898	1421 (58.3)	1428 (41.2)	< 0.001
Concentric hypertrophy–no. (%)	5898	688 (28.2)	1240 (35.8)	< 0.001
Eccentric hypertrophy–no. (%)	5898	733 (30.1)	188 (5.4)	< 0.001
Right atrial size (cm)	5002	5.5 ± 0.9	5.5 ± 0.9	0.39
Right ventricular basal diastolic diameter (cm)	1018	4.2 ± 0.9	3.9 ± 0.9	< 0.001
Peak Doppler transaortic velocity (m/s)	4945	1.8 ± 0.9	2.0 ± 0.9	< 0.001
Aortic regurgitation severity–no. (%)	4443			< 0.001
0+		721 (29.6)	1221 (35.3)	
1+		1069 (43.8)	1302 (37.6)	
2+		61 (2.5)	67 (1.9)	
Mitral regurgitation severity—no. (%)	5345			< 0.001
0+		41 (1.7)	133 (3.8)	
1+		1652 (67.8)	2513 (72.6)	
2+		589 (24.2)	406 (11.7)	
Tricuspid regurgitation severity–no. (%)	4055			0.009
0+		< 11	18 (0.5)	
1+		1251 (51.3)	1902 (54.9)	
2+		237 (9.7)	286 (8.3)	
3+		121 (5.0)	127 (3.7)	
4+		43 (1.8)	61 (1.8)	
**Comorbidities**				
Elixhauser score	5894	9.6 ± 8.6	11.1 ± 9.1	< 0.001
Atrial fibrillation–no. (%)	5898	177 (7.3)	295 (8.5)	0.09
Valvular disease–no. (%)	5894	498 (20.4)	785 (22.7)	0.04
Hypertension—no. (%)	5894	1867 (76.6)	2764 (79.8)	0.003
Diabetes Mellitus—no. (%)				
Uncomplicated	5894	945 (38.8)	1242 (35.9)	0.02
Complicated	5894	522 (21.4)	678 (19.6)	0.09
Renal failure—no. (%)	5894	976 (40.0)	1300 (37.6)	0.05
Pulmonary circulation disorders—no. (%)	5894	134 (5.5)	391 (11.3)	< 0.001
Peripheral vascular disorders–no. (%)	5894	464 (19.0)	548 (15.8)	0.0014
Paralysis—no. (%)	5894	90 (3.7)	115 (3.3)	0.47
Chronic Lung disease—no. (%)	5894	760 (31.2)	1296 (37.4)	< 0.001
Neurologic disorders—no. (%)	5894	209 (8.6)	356 (10.3)	0.03
Hypothyroidism—no. (%)	5894	328 (13.5)	619 (17.9)	< 0.001
Liver disease—no. (%)	5894	113 (4.6)	197 (5.7)	0.08
Peptic Ulcer disease–no. (%)	5894	11 (0.5)	23 (0.7)	0.38
AIDS–no. (%)	5894	< 11	13 (0.4)	0.83
Lymphoma–no. (%)	5894	66 (2.7)	79 (2.3)	0.31
Metastatic cancer–no. (%)	5894	56 (2.3)	112 (3.3)	0.04
Solid tumor without metastasis—no. (%)	5894	126 (5.2)	197 (5.7)	0.42
Rheumatoid arthritis / Collagen Vascular Disorders–no. (%)	5894	97 (4.0)	172 (5.0)	0.08
Coagulopathy—no. (%)	5894	310 (12.7)	438 (12.7)	0.94
Obesity—no. (%)	5894	170 (7.0)	485 (14.0)	< 0.001
Weight loss—no. (%)	5894	136 (5.6)	249 (7.2)	0.01
Fluid and Electrolyte Disorders.–no. (%)	5894	1057 (43.4)	1663 (48.0)	< 0.001
Blood loss anemia—no. (%)	5894	73 (3.0)	143 (4.1)	0.02
Deficiency anemia—no. (%)	5894	695 (28.5)	1187 (34.3)	< 0.001
Alcohol Abuse—no. (%)	5894	24 (1.0)	27 (0.8)	0.48
Drug Abuse—no. (%)	5894	< 11	29 (0.8)	< 0.001
Psychosis—no. (%)	5894	76 (3.1)	188 (5.4)	< 0.001
Depression—no. (%)	5894	257 (10.5)	501 (14.5)	< 0.001

^a^Estimates are presented as means ± standard deviations unless otherwise indicated. Cell numbers < 11 are omitted per Medicare data use policy. N obs = number of observations.

Of those included, 2454 (41.6%) were readmitted for heart failure (HF+) and 3446 (58.4%) were not admitted for heart failure (HF-) at one year. Those readmitted for heart failure (HF+) versus those not-readmitted (HF-) were slightly older, more frequently an inpatient at the time of their index TTE, had a higher NT-proBNP measurement, greater LV hypertrophy, more advanced diastolic dysfunction, a higher E/e’ ratio, larger right ventricular basal diameters, larger left atrial sizes, and greater degrees of mitral, and tricuspid regurgitation on their index TTE (all p < 0.05; **S1 Table in S1 File**). Additionally, those readmitted had more comorbidities (HF+ vs. HF-; mean Elixhauser score, 11.4 ± 9.1 vs. 9.8 ± 8.8 p < 0.001), in particular renal failure, hypertension, and diabetes.

Among the HFrEF subgroup, the top 5 significant variables by standardized univariate effect sizes were drug abuse, inpatient status, hypertension, hypothyroidism, and right ventricular basal diastolic diameter (**Table 2**). Among the HFpEF subgroup, the top 5 significant variables by standardized univariate effect sizes were inpatient status, right ventricular basal diastolic diameter, hypertension, blood loss anemia, and pulmonary circulatory disorders.

**Table 2:** Standardized univariate effect sizes for prediction of heart failure readmission by presence or absence of reduced left ventricular systolic function.

	Heart Failure with Reduced Ejection Fraction (N = 2438)		Heart Failure with Preserved Ejection Fraction (N = 3462)		p-value for interaction
Variable	Unadjusted OR (95% CI)^a^	p-value	Unadjusted OR (95% CI)^a^	p-value	
**Demographic and Physiologic Variables**					
Age	1.11 (1.02–1.21)	0.02	1.08 (1.01–1.16)	0.03	0.64
Female	1.06 (0.92–1.22)	0.39	1.06 (0.90–1.25)	0.48	0.99
Systolic BP	1.01 (0.94–1.08)	0.88	0.99 (0.91–1.09)	0.88	0.84
Diastolic BP	1.03 (0.95–1.12)	0.45	0.97 (0.91–1.04)	0.42	0.28
Heart rate	1.01 (0.93–1.09)	0.89	0.97 (0.81–1.04)	0.38	0.50
Height in cm	1.00 (0.92–1.09)	0.94	1.00 (0.93–1.07)	0.96	0.93
Weight in kg	1.06 (0.98–1.15)	0.15	1.08 (1.01–1.16)	0.03	0.74
Inpatient status	1.86 (1.52–2.27)	< 0.001	1.71 (1.45–2.02)	< 0.001	0.55
NT-proBNP	1.01 (0.91–1.14)	0.79	1.17 (1.07–1.28)	< 0.001	0.06
**Echocardiographic Diastolic Function Variables**					
Mitral valve peak E-wave velocity	1.17 (1.06–1.30)	0.002	1.23 (1.13–1.33)	< 0.001	0.49
Mitral valve peak A wave velocity	0.96 (0.87–1.05)	0.37	0.96 (0.87–1.06)	0.41	0.96
Mitral valve E/A ratio	1.16 (1.05–1.28)	0.003	1.16 (1.05–1.27)	0.003	0.97
Mitral valve deceleration time	0.91 (0.82–1.00)	0.05	0.97 (0.90–1.05)	0.47	0.27
Lateral Mitral E’ Velocity	1.09 (0.87–1.35)	0.46	0.98 (0.89–1.08)	0.72	0.42
Septal Mitral E’ Velocity	0.89 (0.71–1.12)	0.32	0.99 (0.91–1.07)	0.74	0.41
Pulmonary vein S wave velocity (m/s)	0.61 (0.45–0.84)	0.002	0.94 (0.83–1.08)	0.35	0.01
Pulmonary vein D wave velocity (m/s)	0.97 (0.80–1.17)	0.74	1.25 (1.04–1.49)	0.006	0.03
Pulmonary vein S/D ratio	0.72 (0.56–0.92)	0.008	0.78 (0.62–0.96)	0.02	0.67
Pulmonary vein Ar wave velocity (m/s)	0.55 (0.29–1.07)	0.08	1.11 (0.91–1.36)	0.30	0.05
E/e’ ratio	1.24 (1.10–1.39)	0.004	1.21 (1.08–1.35)	< 0.001	0.79
Left atrial size (cm)					0
Superoinferior	1.12 (1.03–1.22)	0.01	1.23 (1.14–1.33)	< 0.001	0.12
Anteroposterior	1.10 (1.01–1.20)	0.04	1.29 (1.19–1.90)	< 0.001	0.007
Left atrial volume index (cm^3^)	1.24 (0.94–1.65)	0.13	1.21 (0.95–1.53)	0.13	0.87
Peak tricuspid regurgitant gradient (mmHg)	1.15 (1.04–1.27)	0.008	1.22 (1.13–1.32)	< 0.001	0.33
Diastolic grade					
None or Grade I	Ref	Ref	Ref	Ref	0.21
Grade II/II	0.95 (0.73–1.24)	0.73	1.19 (0.95–1.50)	0.13	
**Echocardiographic Structural Variables**					
Left ventricular systolic dimension (cm)	1.16 (1.00–1.33)	0.04	1.07 (0.90–1.27)	0.44	0.48
Left ventricular diastolic dimension (cm)	1.12 (1.02–1.22)	0.01	1.09 (0.99–1.20)	0.08	0.71
Left ventricular septal wall thickness (cm)	1.06 (0.97–1.15)	0.21	1.20 (1.11–1.30)	< 0.001	0.03
Left ventricular posterior wall thickness (cm)	1.07 (0.98–1.17)	0.13	1.19 (1.09–1.29)	< 0.001	0.11
Relative wall thickness	1.02 (0.90–1.16)	0.75	1.07 (0.98–1.17)	0.14	0.55
Left ventricular mass (g)	1.14 (1.05–1.24)	0.002	1.22 (1.12–1.33)	< 0.001	0.28
Left ventricular mass index (g/m^2^)	1.14 (1.04–1.24)	0.004	1.15 (1.07–1.24)	0.002	0.80
Left ventricular hypertrophy	1.31 (1.11–1.54)	0.0011	1.14 (0.99–1.31)	0.06	0.20
Concentric hypertrophy	1.12 (0.94–1.34)	0.20	1.13 (0.98–1.30)	0.10	0.96
Eccentric hypertrophy	1.22 (1.03–1.45)	0.02	1.08 (0.80–1.46)	0.61	0.49
Right atrial size (cm)	1.09 (1.00–1.18)	0.06	1.24 (1.15–1.34)	< 0.001	0.02
Right ventricular basal diastolic diameter (cm)	1.37 (1.12–1.68)	0.002	1.55 (1.29–1.87)	< 0.001	0.39
Peak Doppler transaortic velocity (m/s)	1.09 (1.00–1.19)	0.05	1.20 (1.12–1.29)	< 0.001	0.10
Aortic regurgitation severity					
0+	Ref	Ref	Ref	Ref	Ref
1+	0.93 (0.77–1.13)	0.47	(0.85–1.18)	0.96	0.04
2+	0.74 (0.43–1.26)	0.26	1.58 (0.95–2.58)	0.07	0.13
Mitral regurgitation severity					
0+	Ref	Ref	Ref	Ref	Ref
1+	0.88 (0.46–1.64)	0.70	1.12 (0.78–1.61)	0.54	0.98
2+	0.91 (0.48–1.72)	0.70	1.46 (0.97–1.61)	0.07	0.98
Tricuspid regurgitation severity					
0+	Ref	Ref	Ref	Ref	Ref
1+	1.41 (0.34–5.93)	0.64	2.07 (0.68–6.32)	0.20	0.61
2+	1.71 (0.40–7.31)	0.47	3.04 (0.98–9.47)	0.05	0.75
3+	1.26 (0.29–5.50)	0.76	2.51 (0.78–8.04)	0.12	0.50
4+	1.92 (0.41–9.05)	0.41	4.41 (1.30–14.94)	0.02	0.37
**Comorbidities**					
Elixhauser Score	1.20 (1.08–1.32)	< 0.001	1.28 (1.19–1.38)	< 0.001	0.31
Atrial fibrillation	1.00 (0.74–1.36)	0.99	1.26 (0.99–1.60)	0.06	0.25
Valvular disease	1.22 (1.00–1.49)	0.046	1.32 (1.12–1.55)	< 0.001	0.55
Hypertension	1.44 (1.19–1.74)	< 0.001	1.51 (1.26–1.80)	< 0.001	0.73
Diabetes					
Uncomplicated	1.39 (1.18–1.64)	< 0.001	1.36 (1.18–1.56)	< 0.001	0.82
Complicated	1.16 (0.95–1.40)	0.14	1.26 (1.06–1.49)	0.008	0.52
Renal failure	1.28 (1.09–1.51)	0.003	1.37 (1.19–1.58)	< 0.001	0.54
Pulmonary circulatory disorders	1.19 (0.84–1.69)	0.32	1.44 (1.17–1.78)	< 0.001	0.36
Peripheral vascular disorders	1.03 (0.84–1.26)	0.76	1.29 (1.07–1.55)	0.007	0.11
Paralysis	1.08 (0.71–1.65)	0.70	0.75 (0.50–1.11)	0.15	0.21
Chronic Lung Disease	1.29 (1.08–1.53)	0.004	1.27 (1.10–1.46)	0.008	0.91
Neurologic disorders	1.01 (0.76–1.34)	0.97	0.86 (0.68–1.08)	0.19	0.39
Hypothyroidism	1.44 (1.14–1.82)	0.002	1.15 (0.96–1.37)	0.13	0.13
Liver disease	1.05 (0.72–1.53)	0.81	0.89 (0.66–1.20)	0.44	0.51
Peptic ulcer disease	0.44 (0.12–1.67)	0.23	0.44 (0.16–1.19)	0.10	0.99
AIDS	1.18 (0.30–4.74)	0.81	1.36 (0.46–4.06)	0.58	0.88
Lymphoma	0.87 (0.53–1.42)	0.58	0.82 (0.51–1.31)	0.41	0.87
Metastatic cancer	0.50 (0.34–1.05)	0.07	0.55 (0.36–0.84)	0.006	0.79
Solid malignancy	1.08 (0.75–1.55)	0.68	0.89 (0.66–1.20)	0.44	0.42
Rheumatoid arthritis / Collagen Vascular Diseases	0.98 (0.65–1.48)	0.93	1.27 (0.93–1.73)	0.13	0.32
Coagulopathy	0.96 (0.75–1.21)	0.71	1.00 (0.81–1.23)	0.98	0.79
Obesity	1.33 (0.97–1.81)	0.08	1.16 (0.95–1.41)	0.14	0.47
Weight loss	0.77 (0.54–1.09)	0.14	0.77 (0.58–1.01)	0.06	0.99
Electrolyte Abnormality	0.97 (0.83–1.14)	0.73	1.20 (1.05–1.38)	0.009	0.05
Blood loss anemia	1.54 (0.96–2.45)	0.07	1.51 (1.08–2.11)	0.02	0.95
Deficiency Anemias	1.06 (0.89–1.27)	0.49	1.25 (1.08–1.44)	0.002	0.17
Alcohol abuse	1.18 (0.53–2.65)	0.68	0.45 (0.18–1.12)	0.09	0.12
Drug abuse	3.56 (0.37–34.23)	0.27	1.29 (0.62–2.70)	0.49	0.41
Psychoses	1.13 (0.71–1.78)	0.61	0.89 (0.66–1.21)	0.48	0.41
Depression	1.25 (0.96–1.62)	0.09	1.08 (0.89–1.31)	0.46	0.36

^a^Represents the odds ratio (OR) for presence or absence of a dichotomous variable or a 1 standard deviation increase in the predictor for continuous variables

Several variables had larger univariate effect sizes in the HFpEF population including pulmonary vein D-wave velocity (interaction p = 0.03), pulmonary vein Ar velocity (interaction p = 0.05), left atrial anteroposterior dimension (interaction p = 0.007), right atrial size (interaction p = 0.02), left ventricular septal wall thickness (interaction p = 0.03), fluid and electrolyte abnormalities (interaction p = 0.05), and NT-proBNP (interaction p = 0.06). Pulmonary vein S-wave velocity (interaction p = 0.01) hard a larger effect in the HFrEF population.

**Fig 2:**
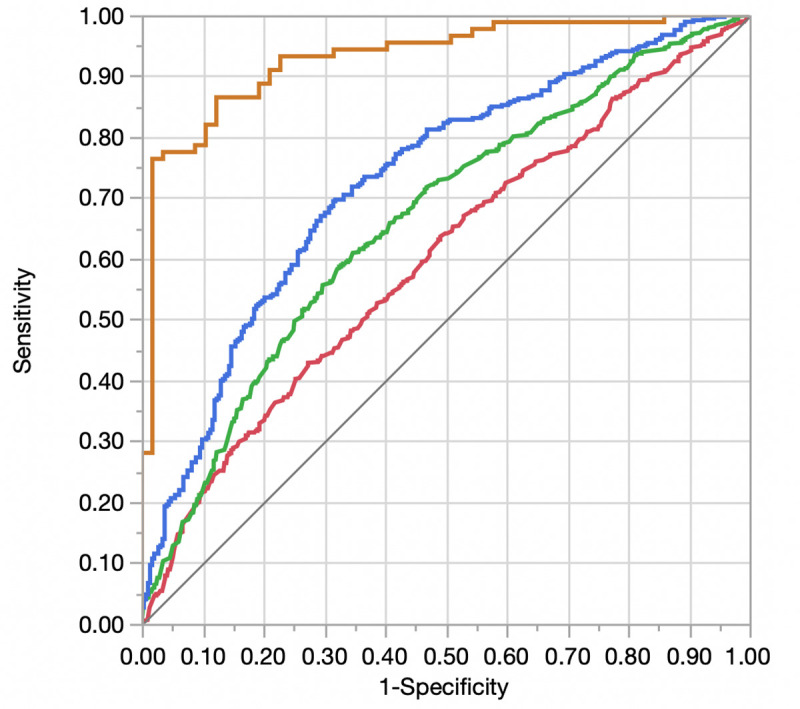
Receiver operator curve displaying differences in discrimination between adjusted models for heart failure readmission. Red = Model 1 (Demographics and Physiologic Variables); Green = Model 2 (Model 1 + Comorbidities); Blue = Model 3 (Model 2 + Echocardiographic Structural Variables); Orange = Model 4 (Model + Echocardiographic Diastolic Variables).

The overall dataset was randomly split into a 50% derivation cohort (N = 2950) and a 50% validation cohort (N = 2950). No variables were significantly different between derivation and validation cohorts. Starting with the base model with physiologic and demographic variables, the addition of comorbidities (Model 2), structural variables (Model 3), and diastolic functional variables (Model 4) was associated with improved model discrimination (**Table 3**). **Fig 2** compares the respective ROC curves for models 1–4. Five variables remained significantly associated with HF hospitalization in the final multivariable model (Model 4): inpatient status, peak tricuspid regurgitant gradient, NT-proBNP, history of solid malignancy, and history of rheumatoid arthritis or collagen vascular disorders (**S2 Table in S1 File**).

**Table 3:** Comparison of nested logistic regression models to predict heart failure readmission in derivation and validation samples.

Model	AUC Derivation (95% CI)^a^	p-value for difference in AUC values (Derivation)	AUC Validation (95% CI)^a^	p-value for difference in AUC values (Validation)
Model 1 (Demographic and Physiologic Variables)	0.60 (0.57–0.63)	Ref	0.58 (0.55–0.61)	Ref
Model 2 (Model 1 + Comorbidities)	0.66 (0.63–0.69)	< 0.001 (Model 2 vs. Model 1)	0.60 (0.57–0.63)	0.08 (Model 2 vs. Model 1)
Model 3 (Model 2 + Echocardiographic Structural Variables)	0.73 (0.69–0.77)	< 0.001 (Model 3 vs. Model 2)	0.61 (0.57–0.65)	0.07 (Model 3 vs. Model 2)
Model 4 (Model 3 + Echocardiographic Diastolic Variables)	0.93 (0.88–0.96)	< 0.001 (Model 4 vs. Model 3)	0.63 (0.54–0.71)	0.61 (Model 4 vs. Model 3)

^**a**^Represents the area under the curve (AUC) for models 1–4 in the derivation (“Derivation”) and validation (“Validation”) samples.

In both the HFrEF (validation AUC [95% CI] in Model 4 = 0.64 [0.43–0.80]) and HFpEF (validation AUC [95% CI] in Model 4 = 0.63 [0.52–0.73]) groups, prediction of HF hospitalization remained poor despite small improvements in model AUC with the addition of comorbidity, echocardiographic structural, and echocardiographic diastolic variables (**S3 and S4 Tables in S1 File**). The AUC in the full model was similar in HFpEF and HFrEF (p for difference in AUC = 0.93).

### Sensitivity analysis

To account for the competing risk of death, analyses were repeated using a composite outcome of death or HF readmission within 1-year after the index TTE. A total of 3617 individuals (61.3% of total) were readmitted for HF or died within 1-year of TTE. In the overall sample, addition of comorbidity and structural to the base model (e.g. Model 1) resulted in incremental improvement in discrimination but resulted in model overfitting and loss of discrimination in the validation sample with the addition of diastolic variables (**S5 Table and S1 Fig in S1 File**). In both HFrEF (**S6 Table in S1 File**) and HFpEF (**S7 Table in S1 File**), discrimination of death or HF readmission at 1-year was numerically higher but statistically similar to that of HF readmission alone (HFrEF p = 0.58; HFpEF p = 0.44). Similar to the primary endpoint, the AUC of the fully adjusted model (Model 4) using the composite endpoint was not different between the HFrEF and HFpEF subgroups (p = 0.16). The AUC of the fully adjusted model (Model 4) using the endpoint of HF readmission within 1-year was not different in outpatients vs. inpatients (p = 0.64; **S8 and S9 Tables in S1 File)**.

## Discussion

Although HF readmission is a common and important source of morbidity and costs in individuals with pre-existing HF [30], prediction of an individual’s likelihood of HF readmission remains difficult using conventional parameters. In this study of individuals undergoing TTE at a single, large academic medical center, we found that addition of echocardiographic diastolic variables to echocardiographic structural variables and comorbidities improved prediction of HF readmission, regardless of LVEF, though prediction accuracy remained modest. LV wall thickness, atrial dimensions, NT-proBNP, and pulmonary vein S-wave and Ar-wave velocities predicted HF readmission in HFpEF but not HFrEF. The additional role of medication adherence, readmission history, and functional status in differential prediction of HF readmission by LVEF category should be considered for future study.

Our study confirms results of prior work evaluating the incremental ability of echocardiographic variables to predict HF readmission in HFpEF. In the Heart and Soul study, presence of diastolic dysfunction, left atrial volume index, left ventricular mass index, mitral regurgitation, and left ventricular outflow tract velocity-time integral predicted future HF events in individuals with stable coronary artery disease [8, 16]. While this analysis was adjusted for LVEF, 92.7% of participants had an LVEF > 45% at baseline. In a separate study (in which 65% of the population had an LVEF ≥50%), left atrial volume index and E/e’ were predictive of subsequent HF readmission independent of baseline LVEF and LVEDP [17]. In a prospective HFpEF registry, only moderate to severe diastolic dysfunction and a high number (≥4) of abnormal diastolic parameters were associated with worse prognosis over short term follow-up (i.e. 4–8 weeks) [18]. In an echocardiographic substudy of the Angiotensin-Neprilysin Inhibition in Heart Failure with Preserved Ejection Fraction (PARAGON-HF) trial, wall thickness, mass, E/e’ ratio, tricuspid regurgitation velocity, and enlarged right ventricular size were associated with HF readmission or cardiovascular death [31]. In the current study, specific parameters such as wall thickness, atrial dimensions, NT-proBNP, and pulmonary vein D-wave and Ar-wave velocities better predicted HF readmission in HFpEF than HFrEF. Worsening diastolic grade was associated with a trend towards increased HF readmission risk in HFpEF that did not meet statistical significance. Nevertheless, despite the moniker of HFpEF, diastolic parameters as a whole were equally and incrementally predictive of outcomes regardless of systolic function.

While less well studied, the current study also confirms the importance of LV dimensions in HFrEF. Using data from the multinational observational BIOSTAT-CHF study, Nauta et al. demonstrated that HFrEF patients with eccentric hypertrophy may have greater a greater mortality benefit from uptitration of angiotensin-converting enzyme inhibitors / angiotensin receptor blockers and beta-blockers compared to HFrEF patients with concentric hypertrophy [22]. Interestingly, although the interaction p-value was nonsignificant, both eccentric hypertrophy and left ventricular dimensions were significantly predictive of HF risk in HFrEF but not HFpEF.

The current study differs from many prior studies by enrolling a predominantly mixed systolic function cohort. In doing so, we were able to evaluate the differential effects of echocardiographic variables across systolic function groups. Thus, while we confirm that LV dimensions and eccentric hypertrophy are important prognosticators in HFrEF, we find that they are not (despite adequate statistical power for resolution) predictive of readmission in HFpEF. Similarly, we found that wall thickness measures were not independently prognostic in HFrEF, despite being prognostic in HFpEF. Taken together, these findings suggest that predictive models of HF readmission weight the relative importance of some variables differently depending on the LVEF category under consideration. Moreover, these findings suggest that future studies should consider evaluating mixed systolic function cohorts as it should not be assumed that findings generalize across LV function categories [32]. While the concept that predictors of HF hospitalization may differ between HFrEF and HFpEF is physiologically evident, these data suggest that these differences may be of predictive relevance as well.

Regardless of the group evaluated or variables used, discrimination of HF hospitalization was only modest, similar to prior studies, and numerically less predictive (though not statistically different) than the composite of HF hospitalization or death. Thus, our study suggests that one possible reason for the modest predictive ability of current models for HF readmission may be the high mortality rate in this population. A predictive model that considers the risk of HF readmission alone may underperform a model that accounts for the competing risk of mortality in estimating risk of readmission.

The current study also confirms the incremental value of imaging variables to clinical variables in predicting HF risk across LVEF categories. Insofar as imaging parameters contain information on an individual’s risk of having a given outcome that is orthogonal to existing information in the risk model, the addition of imaging data can greatly assist in understanding unaccounted-for variation between individuals in their risk of an outcome. Such multi-parametric approaches to prediction integrating multiple variables from several different sources (e.g. biomarkers, imaging markers, clinical data) may improve upon single-parametric approaches in predicting future HF readmission, mortality, and even response to HF therapy [23]. In the current study, the biomarker, NT-proBNP, was among the most predictive variables for HF readmission in the HFpEF cohort, suggesting the value of including multiparametric data in prediction algorithms. To this end, it remains uncertain how novel echocardiographic parameters such as LV global longitudinal strain and left atrial strain perform in predicting HF readmission, though these may also augment prediction ability compared to conventional measures [33, 34]. Furthermore, while not evaluated in the current study, the additional role of medication adherence, readmission history, and functional status in differential prediction of HF readmission by LVEF category should be considered for future study. Nevertheless, the current study underlies the need for consideration of LV remodeling in prediction of future HF readmission and the additive benefits of including diastolic functional variables in prognostication [35].

There are several limitations to the current study. First, as a single center retrospective analysis, albeit large, results may not generalize to other sites and may subject to residual confounding. Findings may not generalize beyond one-year follow-up and do not reflect subsequent changes in LVEF on risk of HF hospitalization. Additionally, it is possible that echocardiographic variables may be more predictive of heart failure hospitalizations closer to the date of the index echocardiogram (e.g. 3–6 months). While the time horizon of the current study includes heart failure hospitalizations occurring within 3–6 months in the 1-year endpoint, prediction accuracy may differ. Additionally, as the principal scientific question involved the binary presence or absence of HF readmission at 1-year, time-to-event analyses were not used. Thus, the parameters evaluated could have differential effects on timing of events in HFpEF vs. HFrEF. As diastolic grade was retrospectively applied, it is possible that the diastolic grading used may differ from that reported in clinical interpretation. Importantly, data on medication use or adherence, readmission status, and New York Heart Association class were not available which may contribute to the modest predictive accuracy of echocardiographic and clinical parameters, and should be considered in future studies on this topic. Moreover, as evidence-based therapies that modify outcomes are not currently available for HFpEF, it is likely that inclusion of medications will modify the predictive accuracy of HFrEF models to a greater extent than HFpEF, though should be tested in future study.

## Conclusions

Among elderly individuals with heart failure in this single-center retrospective analysis, the addition of echocardiographic structural and diastolic variables augmented prediction of HF readmission compared with comorbidities alone, regardless of LVEF, though predictive accuracy remained modest. Certain structural variables, but not all, were solely predictive of readmission in one LVEF category. Thus, future HF readmission models should account for these differential variable effects and competing risk to obtain accurate predictions of risk, though further study is needed of the additional role of medication adherence, readmission history, and functional status on differential prediction of heart failure rehospitalization.

## Supporting information

S1 File (PDF)
