# Supplementary material for: Retrospective evaluation of echocardiographic variables for prediction of heart failure hospitalization in heart failure with preserved versus reduced ejection fraction: A single center experience
Source: PLoS One. 2020 Dec 22;15(12):e0244379. doi: 10.1371/journal.pone.0244379 (PMC7755281; doi:10.1371/journal.pone.0244379)
Supplement: S1 File — (PDF) [file pone.0244379.s001.pdf]

SUPPORTING INFORMATION

S1 Table 1. Baseline characteristics of included patients by presence or absence of heart failure hospitalization within 1 year of the index echocardiogram

	N obs	Any heart failure hospitalization within 1 year of index echocardiogram		P-value
		Yes<br>(N = 2454)	No<br>(N = 3446)	
**Demographic and Physiologic Variables**				
Age (years)	5900	77.4 ± 10.7	76.6 ± 11.1	0.003
Female – no. (%)	5900	1201 (48.9)	1693 (49.1)	0.89
Systolic BP (mmHg)	5763	126 ± 33	127 ± 34	0.62
Diastolic BP (mmHg)	5751	68 ± 37	68 ± 37	0.82
Heart rate (bpm)	5447	76 ± 20	76 ± 21	0.74
Height (cm)	5657	167 ± 12	167 ± 12	0.33
Weight (kg)	5731	82 ± 24	80 ± 24	0.02
Body Mass Index – (kg/m^2)	5628	29.6 ± 15.1	28.9 ± 9.0	0.046
Inpatient at the time of echocardiogram – no. (%)	5900	2026 (82.6)	2504 (72.7)	< 0.001
NT-proBNP – median (IQR)	3142	4856 (1884-11624)	3175 (1165-8723)	< 0.001
**Echocardiographic Diastolic Function Variables**				
Mitral valve peak E-wave velocity (m/s)	5102	1.0 ± 0.3	1.0 ± 0.4	< 0.001
Mitral valve peak A wave velocity (m/s)	3800	0.9 ± 0.4	0.9 ± 0.4	0.049
Mitral valve E/A ratio	3795	1.4 ± 0.9	1.3 ± 0.9	< 0.001
Mitral valve deceleration time (ms)	4866	209 ± 72	215 ± 76	0.004
Lateral Mitral e' Velocity (cm/s)	2969	8.7 ± 12.8	8.7 ± 21.6	0.88
Septal Mitral e' Velocity (cm/s)	2931	6.1 ± 4.1	6.5 ± 12.8	0.32
Pulmonary vein S wave velocity (m/s)	1418	0.5 ± 0.3	0.6 ± 0.4	0.03
Pulmonary vein D wave velocity (m/s)	1298	0.5 ± 0.3	0.5 ± 0.4	0.16
Pulmonary vein S/D ratio	1292	1.1 ± 0.6	1.3 ± 1.1	0.002
Pulmonary vein Ar wave velocity (m/s)	764	0.3 ± 0.3	0.3 ± 0.1	0.59
E/e' ratio	2855	15.6 ± 6.8	14.2 ± 6.4	< 0.001
Left atrial size (cm)<br>Superoinferior<br>Anteroposterior	 5042<br>5248	 5.9 ± 0.9<br>4.5 ± 0.8	5.<br>5.8 ± 0.9<br>4.4 ± 0.8	 < 0.001<br>< 0.001
Left atrial volume index (cm^3/m^2)	551	40.2 ± 11.7	37.9 ± 11.2	0.03
Peak estimated pulmonary artery systolic pressure (mmHg)	4509	37.5 ± 13.6	35.4 ± 12.8	< 0.001

Diastolic Grade – no. (%)	5900			0.048
Normal/Grade I		2076 (84.6)	2993 (86.9)	
Grade II		291 (11.9)	347 (10.1)	
Grade III		87 (3.6)	106 (3.1)	
**Echocardiographic Structural Variables**				
Left ventricular ejection fraction (%)	5900	50.4 ± 20.6	53.4 ± 20.0	< 0.001
Left ventricular systolic dimension (cm)	3039	3.4 ± 1.2	3.2 ± 1.0	< 0.001
Left ventricular diastolic dimension (cm)	5238	4.9 ± 0.9	4.8 ± 0.9	< 0.001
Left ventricular septal wall thickness (cm)	5193	1.2 ± 0.2	1.2 ± 0.2	< 0.001
Left ventricular posterior wall thickness (cm)	5174	1.2 ± 0.2	1.1 ± 0.2	0.003
Relative wall thickness	5132	0.50 ± 0.16	0.50 ± 0.16	0.44
Left ventricular mass (g)	5131	228 ± 77	214 ± 72	< 0.001
Left ventricular mass index (g/m^2)	4965	119 ± 40	113 ± 36	< 0.001
Left ventricular hypertrophy – no. (%)	5898	1268 (51.7)	1581 (45.9)	< 0.001
Concentric hypertrophy – no. (%)	5898	831 (33.9)	1097 (31.8)	< 0.001
Eccentric hypertrophy – no. (%)	5898	437 (17.8)	484 (14.0)	< 0.001
Right atrial size (cm)	5002	5.6 ± 0.9	5.5 ± 0.9	< 0.001
Right ventricular basal diastolic diameter (cm)	1018	4.2 ± 1.0	3.9 ± 0.9	< 0.001
Peak Doppler transaortic velocity (m/s)	4945	2.0 ± 1/0	1.9 ± 0.9	< 0.001
Aortic regurgitation severity – no. (%)	4443			0.77
0+		803 (32.7)	1140 (33.1)	
1+		980 (39.9)	1392 (40.4)	
2+		57 (2.3)	71 (2.1)	
Mitral regurgitation severity - no. (%)	5345			0.04
0+		67 (2.7)	107 (3.1)	
1+		1710 (69.7)	2457 (71.3)	
2+		454 (18.5)	541 (15.7)	
Tricuspid regurgitation severity – no. (%)	4055			< 0.001
0+		< 11	19 (0.6)	
1+		1281 (52.2)	1873 (54.4)	
2+		253 (10.3)	270 (7.8)	
3+		105 (4.3)	143 (4.1)	
4+		57 (2.3)	47 (1.4)	
**Comorbidities**				
Elixhauser score	5894	11.4 ± 9.1	9.8 ± 8.8	< 0.001
Atrial fibrillation – no. (%)	5898	210 (9.0)	262 (7.6)	0.19
Valvular disease – no. (%)	5894	592 (24.1)	691 (20.1)	< 0.001
Hypertension - no. (%)	5894	2015 (82.1)	2618 (76.0)	< 0.001
Diabetes Mellitus - no. (%)		0.		
Uncomplicated	5894	1019 (41.5)	1169 (33.9)	< 0.001
Complicated	5894	546 (22.2)	654 (19.0)	0.002
Renal failure - no. (%)	5894	1046 (42.6)	1230 (35.7)	< 0.001
Pulmonary circulation disorders - no. (%)	5894	249 (10.1)	276 (8.0)	0.005
Peripheral vascular disorders – no. (%)	5894	456 (18.6)	557 (16.2)	0.02
Paralysis - no. (%)	5894	80 (3.3)	125 (3.6)	0.47

Chronic Lung disease - no. (%)	5894	928 (37.8)	1128 (32.7)	< 0.001
Neurologic disorders - no. (%)	5894	222 (9.0)	343 (10.0)	0.24
Hypothyroidism - no. (%)	5894	432 (17.6)	515 (14.9)	0.007
Liver disease - no. (%)	5894	124 (5.1)	186 (5.4)	0.59
Peptic Ulcer disease – no. (%)	5894	< 11	26 (0.8)	0.04
AIDS – no. (%)	5894	< 11	11 (0.3)	0.66
Lymphoma – no. (%)	5894	55 (2.2)	90 (2.6)	0.39
Metastatic cancer – no. (%)	5894	48 (2.0)	120 (3.5)	< 0.001
Solid tumor without metastasis - no. (%)	5894	131 (5.3)	192 (5.6)	0.73
Rheumatoid arthritis / Collagen Vascular Disorders – no. (%)	5894	120 (4.9)	149 (3.7)	0.31
Coagulopathy - no. (%)	5894	308 (12.6)	440 (12.8)	0.81
Obesity - no. (%)	5894	291 (11.9)	364 (10.6)	0.12
Weight loss - no. (%)	5894	136 (5.5)	249 (7.2)	0.01
Fluid and Electrolyte Disorders. – no. (%)	5894	1160 (47.3)	1560 (45.3)	0.14
Blood loss anemia - no. (%)	5894	110 (4.5)	106 (3.1)	0.005
Deficiency anemia - no. (%)	5894	826 (33.7)	1056 (30.6)	0.01
Alcohol Abuse - no. (%)	5894	18 (0.7)	33 (1.0)	0.39
Drug Abuse - no. (%)	5894	16 (0.7)	17 (0.5)	0.48
Psychosis - no. (%)	5894	105 (4.3)	159 (4.6)	0.57
Depression - no. (%)	5894	332 (13.5)	427 (12.4)	0.21

Estimates are presented as means ± standard deviations unless otherwise indicated. Cell numbers < 11 are omitted per Medicare data use policy.

S1 Table 2. Variables significantly associated with heart failure readmission at 1-year after multivariable adjustment

Variable	Adjusted OR (95% CI) for Heart Failure Readmission at 1-year	p-value
Inpatient status	2.97 (1.48-5.93)	0.002
Peak tricuspid regurgitant gradient [a]	2.04 (1.34-3.08)	< 0.001
NT-proBNP [a]	1.55 (1.04-2.32)	0.03
Solid malignancy	3.89 (1.06-14.30)	0.04
Collagen vascular diseases / rheumatoid arthritis	3.60 (1.03-12.52)	0.04

Represents the results of a multivariable logistic regression model, adjusting for all variables included in Table 1. [a]Represents the adjusted odds ratio (OR) for a 1-standard deviation increase in the predictor variable.

S1 Table 3. Area Under the Curve for Models to Predict Heart Failure Readmission in the Heart Failure with Reduced Ejection Fraction Subgroup

Model	AUC Derivation (95% CI)[a]	AUC Validation (95% CI)[b]
Model 1 (Demographic and Physiologic Variables)	0.59 (0.55-0.64)	0.56 (0.51-0.61)
Model 2 (Model 1 + Comorbidities)	0.63 (0.59-0.58)	0.57 (0.53-0.62)
Model 3 (Model 2 + Echocardiographic Structural Variables)	0.70 (0.62-0.77)	0.59 (0.51-0.67)
Model 4 (Model 3 + Echocardiographic Diastolic Variables)	0.94 (0.73-0.99)	0.64 (0.43-0.80)

[a]Represents the area under the curve (AUC) for models 1-4 in the derivation sample. [b]Represents the area under the curve (AUC) for models 1-4 in the validation sample.

S1 Table 4. Area Under the Curve for Models to Predict Heart Failure Readmission in the Heart Failure with Preserved Ejection Fraction Subgroup

Model	AUC Derivation (95% CI)[a]	AUC Validation (95% CI)[b]
Model 1 (Demographic and Physiologic Variables)	0.60 (0.56-0.64)	0.58 (0.55-0.62)
Model 2 (Model 1 + Comorbidities)	0.68 (0.64-0.72)	0.61 (0.58-0.65)
Model 3 (Model 2 + Echocardiographic Structural Variables)	0.74 (0.70-0.79)	0.61 (0.56-0.66)
Model 4 (Model 3 + Echocardiographic Diastolic Variables)	0.92 (0.85-0.96)	0.63 (0.52-0.73)

[a]Represents the area under the curve (AUC) for models 1-4 in the derivation sample. [b]Represents the area under the curve (AUC) for models 1-4 in the validation sample.

S1 Table 5. Sensitivity analysis; Comparison of Nested Logistic Regression Models to Predict Heart Failure Readmission or One-Year Mortality in Derivation and Validation Samples

Model	AUC Derivation (95% CI)[a]	p-value for difference in AUC values (Derivation)	AUC Validation (95% CI)[a]	p-value for difference in AUC values (Validation)
Model 1 (Demographic and Physiologic Variables)	0.66 (0.63-0.69)	Ref	0.65 (0.62-0.68)	Ref
Model 2 (Model 1 + Comorbidities)	0.71 (0.68-0.73)	< 0.001 (Model 2 vs. Model 1)	0.66 (0.63-0.69)	0.55 (Model 2 vs. Model 1)
Model 3 (Model 2 + Echocardiographic Structural Variables)	0.78 (0.74-0.81)	< 0.001 (Model 3 vs. Model 2)	0.66 (0.62-0.70)	> 0.99 (Model 3 vs. Model 2)
Model 4 (Model 3 + Echocardiographic Diastolic Variables)	0.98 (0.95-0.99)	< 0.001 (Model 4 vs. Model 3)	0.61 (0.52-0.69)	0.03 (Model 4 vs. Model 3)

[a]Represents the area under the curve (AUC) for models 1-4 in the derivation and validation samples.

S1 Table 6. Sensitivity analysis; Area Under the Curve for Models to Predict Heart Failure Readmission or One-Year Mortality in the Heart Failure with Reduced Ejection Fraction Subgroup

Model	AUC Derivation (95% CI)[a]	AUC Validation (95% CI)[b]
Model 1 (Demographic and Physiologic Variables)	0.64<br>(0.69-0.68)	0.62<br>(0.67-0.68)
Model 2 (Model 1 + Comorbidities)	0.67<br>(0.63-0.72)	0.62<br>(0.57-0.67)
Model 3 (Model 2 + Echocardiographic Structural Variables)	0.79<br>(0.72-0.85)	0.68<br>(0.60-0.75)
Model 4 (Model 3 + Echocardiographic Diastolic Variables)	0.98<br>(0.89-0.99)	0.71<br>(0.53-0.84)

[a]Represents the area under the curve (AUC) for models 1-4 in the derivation sample. [b]Represents the area under the curve (AUC) for models 1-4 in the validation sample.

S1 Table 7. Sensitivity analysis; Area Under the Curve for Models to Predict Heart Failure Readmission or One-Year Mortality in the Heart Failure with Preserved Ejection Fraction Subgroup

Model	AUC Derivation (95% CI)[a]	AUC Validation (95% CI)[b]
Model 1 (Demographic and Physiologic Variables)	0.67 (0.63-0.71)	0.65 (0.61-0.69)
Model 2 (Model 1 + Comorbidities)	0.72 (0.69-0.76)	0.67 (0.63-0.70)
Model 3 (Model 2 + Echocardiographic Structural Variables)	0.78 (0.73-0.81)	0.65 (0.60-0.70
Model 4 (Model 3 + Echocardiographic Diastolic Variables)	0.98 (0.95-0.99)	0.57 (0.47-0.67)

[a]Represents the area under the curve (AUC) for models 1-4 in the derivation sample. [b]Represents the area under the curve (AUC) for models 1-4 in the validation sample.

S1 Table 8. Sensitivity analysis; Area Under the Curve for Models to Predict Heart Failure Readmission from Inpatient Echocardiograms

Model	AUC Derivation (95% CI)[a]	AUC Validation (95% CI)[b]
Model 1 (Demographic and Physiologic Variables)	0.56 (0.52-0.59)	0.54 (0.50-0.57)
Model 2 (Model 1 + Comorbidities)	0.63 (0.60-0.66)	0.56 (0.53-0.60)
Model 3 (Model 2 + Echocardiographic Structural Variables)	0.69 (0.64-0.73)	0.57 (0.52-0.62)
Model 4 (Model 3 + Echocardiographic Diastolic Variables)	0.89 (0.81-0.94)	0.56 (0.44-0.67)

[a]Represents the area under the curve (AUC) for models 1-4 in the derivation sample. [b]Represents the area under the curve (AUC) for models 1-4 in the validation sample.

S1 Table 9. Sensitivity analysis; Area Under the Curve for Models to Predict Heart Failure Readmission from Outpatient Echocardiograms

Model	AUC Derivation (95% CI)[a]	AUC Validation (95% CI)[b]
Model 1 (Demographic and Physiologic Variables)	0.59 (0.52-0.65)	0.49 (0.43-0.55)
Model 2 (Model 1 + Comorbidities)	0.64 (0.57-0.70)	0.59 (0.53-0.64)
Model 3 (Model 2 + Echocardiographic Structural Variables)	0.66 (0.57-0.74)	0.56 (0.48-0.64)
Model 4 (Model 3 + Echocardiographic Diastolic Variables)	0.99 (0.95-0.99)	0.61 (0.42-0.77)

[a]Represents the area under the curve (AUC) for models 1-4 in the derivation sample. [b]Represents the area under the curve (AUC) for models 1-4 in the validation sample.

S1 Fig. Sensitivity analysis; Receiver Operator Curve Displaying Differences in Discrimination between Adjusted Models for Heart Failure Readmission or One-Year Mortality

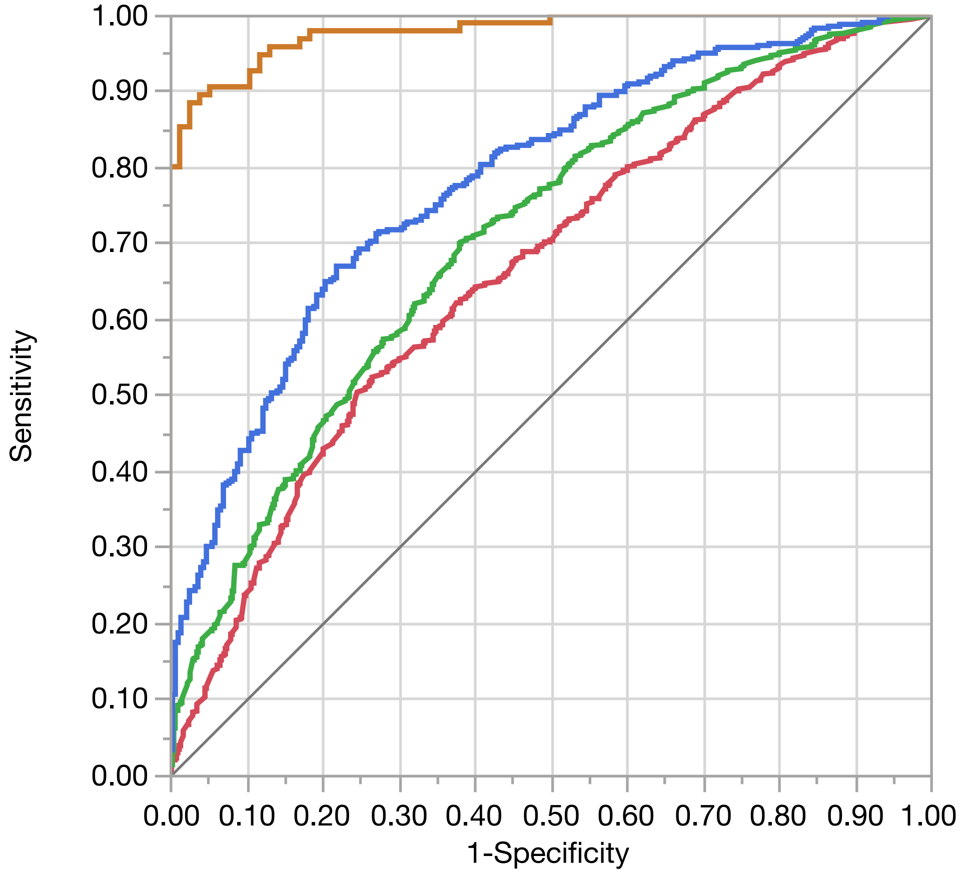

Red = Model 1 (Demographics and Physiologic Variables); Green = Model 2 (Model 1 + Comorbidities); Blue = Model 3 (Model 2 + Echocardiographic Structural Variables); Orange = Model 4 (Model + Echocardiographic Diastolic Variables); $p < 0.0001$ for comparison across models in the derivation sample and $p = 0.20$ in the validation sample.
